# Demographic Characteristics and Parenthood Across Three Cohorts of Sexual Minority Adults

**DOI:** 10.1111/famp.70012

**Published:** 2025-02-18

**Authors:** Kay A. Simon, Gaëlle Meslay, Rachel H. Farr, Stephen T. Russell

**Affiliations:** ^1^ Department of Family Social Science, College of Education and Human Development University of Minnesota Minneapolis Minnesota USA; ^2^ Institut National D'études Démographiques (INED) Paris France; ^3^ Department of Human Development and Family Sciences, School of Human Ecology, College of Natural Sciences University of Texas at Austin Austin Texas USA; ^4^ Department of Psychology, College of Arts and Sciences University of Kentucky Lexington Kentucky USA

**Keywords:** sexual minority adults, sexual minority parent, sexual minority parent demographics

## Abstract

As measurement of sexual minority (SM) people's demographic characteristics has evolved over time, it is of interest to understand how identity intersections within SM communities, such as SM parents, have also changed. The current study aimed to investigate how SM parents may or may not differ in demographic characteristics from SM childfree adults and how the demographic characteristics of SM parents may differ across three cohorts. Participants could be part of one of three age cohorts, with each cohort reflecting distinct historic or cultural events related to LGBTQ+ people's experiences. We used data from a national probability study of 1502 SM adults conducted between 2016 and 2017 to compare demographic characteristics by parent and cohort status. SM parents (*n* = 297) and childfree adults differed in sexual and gender identity, relationship status, educational attainment, urbanicity, and poverty status. There were differences among SM parents based on cohort status in sexual and gender identity, partner status (and gender of the partner), educational attainment, poverty status, and urbanicity. However, there were no differences based on racial/ethnic identity or geographic region among SM parents. This work contributes to the ongoing literature on SM parent families by providing a view of the ways in which SM parents have, and have not, changed demographically over time in the United States.

## 1 Introduction

Historically, sexual minority (SM) people have been barred from parenthood, although research indicates that SM people want to, and do become parents (Riskind and Tornello 2017). It was only in 2015 that marriage for and adoption by same‐sex couples (*Obergefell v. Hodges 2015*) became legally available nationwide in the United States. Assisted reproductive technologies (ART) have also opened the door for more SM adults to become parents. However, SM parents may also experience greater stress (Bos et al. 2016), fewer resources, or available support systems (Reczek 2020), likely due to a legacy of discrimination, compared with their SM childfree and cisgender heterosexual parent counterparts.

Only in recent years has research shown that marriage is a promotive factor among same‐sex couples, although this has been established among different‐sex couples for decades (Reczek 2020). SM parents may be especially vulnerable given minority stressors (i.e., excess stressors due to structural marginalization of one's identity) unique to their SM and parent identities (e.g., stigma from heteronormativity and potential rejection from SM communities due to beliefs that SM identity and parenthood are incompatible; Brooks 1981; Meyer 2003). Furthermore, minority stressors may be exacerbated by tumultuous social and policy changes. With dynamic policy change impacting SM people (e.g., changes in adoption law that superseded the legal benefits of same‐sex marriage; Movement Advancement Project 2023), understanding the sociodemographic characteristics of SM parents and childfree adults, and patterns across cohorts, are important considerations (Reczek 2020). If the background characteristics of SM parents are not well understood, then family policy or cultural competency training for family planning providers aimed at supporting specific groups may be too broad and ineffective. Thus, our goal was to understand and compare differences in sociodemographic backgrounds between SM parents and childfree adults given sociopolitical shifts over time.

### 1.1 Pathways to Parenthood and Policy for SM Parent Families

Family and family planning policy in the United States is often in flux and of particular concern for groups who may be marginalized such as SM adults who want to, or are already, parents (Reczek 2020). There are substantial barriers to parenthood as it relates to SM intended parents such as legal, financial, and cultural barriers, many of which are interwoven concerns (Gato et al. 2017). For example, although sexual intercourse is the most common method of family formation for cisgender different‐sex couples, this does not apply to all SM people (Reczek 2020). Other pathways to parenthood such as adoption or ART may also be out of reach or especially difficult. As it relates to adoption, future parents are often concerned about experiences of discrimination which is warranted given that in some areas of the United States, adoption agencies can legally discriminate against same‐sex couples (Patterson and Farr 2022).

For many SM intended parents, ART may also not be an option due to financial concerns or success rate (i.e., the number of live births following ART). One such contributor is minority stress, as many minoritized groups report lower rates of success and greater stress than their majority group counterparts (Jain et al. 2019). At the intersection of adoption and ART comes additional challenges wherein partners who are non‐biologically related to their children must complete a second‐parent adoption to be granted a legal parenting relationship. It is also relevant to note that these issues do not apply equally to all SM individuals and partners who want to, or become, parents. A substantial number of SM parents identify as bisexual, are in different‐sex relationships, or had children via sexual intercourse and conception (Manley and Ross 2020). With barriers to, and differences in availability of, pathways to parenthood experienced by SM future parents, it is of interest to investigate the differences in background characteristics between groups (SM parents and childfree adults) as well as within groups (among SM parents).

Although there are persistent barriers related to future parenthood among SM people, notable advancements across previous decades have resulted in generational differences. For example, some ARTs such as in vitro fertilization were not available prior to the 1980s (Jain et al. 2019), which may have resulted in an increase in the number of SM parents after that time based on an additional pathway to parenthood (although the total number of SM parents were still decreasing). Other avenues to parenthood have also shifted, including transnational adoption (i.e., adopting a child from another country; Wexler et al. 2023). However, these changes did not uniformly impact all SM future parents. Advancements in ART, but not necessarily surrogacy laws, likely suggest a greater increase in future SM parents who could carry a child (and who could afford ART), rather than all future SM parents (Gates 2015; Smock and Schwartz 2020). Other pathways to parenthood however might have uniformly impacted all SM groups. Although SM different‐sex couples in previous decades may have had the option of transnational adoption (to a greater degree than same‐sex couples), transnational adoption in the United States has seen a steady decline for several reasons. For instance, changes in international law and acknowledgement of transnational adoption as a potential form of settler colonialism (Wexler et al. 2023) have dramatically shaped perceptions of and preferences for adoption as a pathway to parenthood.

Broadly, in previous decades the most common method of becoming an LGBTQ+ parent was having had a child in the context of a previous, different‐sex relationship (and was often assumed to be heterosexual). That is, SM individuals would either not realize they were SM or they formed families while closeted and only later came to openly identify with a SM identity (Gates 2015). This has changed in previous decades given that many individuals in the United States realize their sexual or gender minority identity at younger ages. In turn, family formation in the context of a previous different‐sex relationship has been decreasing, whereas other methods, such as ART have been increasing (Gates 2015). Furthermore, it was only in recent years that LGBTQ+ parenthood was routinely measured in surveys the United States as much of the previous research had to estimate the number of SM parent families in roundabout ways such as assessment of tax records, combining multiple datasets, or same‐sex couple households (Gates 2013), which have limitations in that they do not account for the wide diversity in SM parent families. These changes in access to different family formation methods as well as in family and LGBTQ+ policy over time may have led to shifts in the demographic characteristics of SM parents. However, to our knowledge, research has not comprehensively reported on the potential shifts in demographic characteristics among SM childfree adults and parents.

### 1.2 Demographic Characteristics of SM Parents and Childfree Adults

Research has found several differences in parenthood status among SM people. Specifically, a lower proportion of SM men are parents compared with heterosexual men, and compared with different‐sex parents, a greater proportion of same‐sex parents are people of color, live in the Southern United States, and have a lower household income (Gates 2013). Existing studies also show that more SM people assigned female at birth are parents compared with those assigned male at birth, and that SM parents are more likely to be partnered than childfree SM adults (Goldberg and Kerith 2018). These differences are also relevant given that sex assigned at birth also changes the preferred and possible pathways to parenthood among SM adults.

Although research increasingly attends to intersections among SM populations, such as relationship status (i.e., SM adults in relationships compared with SM single adults) or age (Fredriksen‐Goldsen et al. 2017), less work has focused on the intersection of SM identity and parenthood across a number of demographic characteristics. Furthermore, this work is also often cross‐sectional or limited in other ways, such as only identifying SM parents by nature of same‐sex couple residential status (Gates 2013; Kastanis and Wilson 2014). This approach leaves out SM single parents, SM parents in long‐distance relationships, or those in different‐sex relationships. To fill gaps in the literature, the current study aimed to investigate differences in sociodemographic characteristics between SM parents and childfree adults as well as differences across age cohorts of SM parents. In turn, this work can provide information about the potential changes in sociodemographic characteristics (e.g., changes in proportion of parents of color compared with White parents) of current and future SM parent families (Reczek 2020).

### 1.3 Current Study

The overarching goal of this study was to highlight key differences in sociodemographic characteristics between SM parents and childfree adults and among SM parents themselves as shaped by sociopolitical shifts at different points in history. We used survey‐based data collected as part of the Generations Study, a national probability sample of SM adults (*N* = 1518) across three age cohorts, who were adolescents during specific landmark events (i.e., the Stonewall Riots, AIDS crisis, and same‐sex marriage equality, respectively) that shaped the culture of sexual minorities over the past several decades in the United States. Although our approach was exploratory, we anticipated some variations based on sociodemographic differences, aligned with previous research (Fredriksen‐Goldsen et al. 2017), much of which are age‐related. We expected that SM parents would be older, on average, and more likely to report partners than SM childfree adults. We also anticipated that there would be more SM parents in the older cohorts when compared with the youngest cohort and that they would have greater educational attainment. This anticipation is informed by a lifespan perspective such that, over time, adults have more time to be in relationships as well as to make decisions about family formation and educational attainment.

## 2 Method

### 2.1 Procedure and Participants

The data used here are from the first time point of data collection in a longitudinal national probability study. Participants were recruited through Gallup Inc., a US survey research company between March 2016–March 2017. To recruit SM adults, Gallup Inc. engaged in random digit dialing and inquired as to whether an individual “personally identified as lesbian, gay, bisexual or transgender.” Participants who identified as transgender were redirected to another study, and those who identified as sexual minorities were invited to participate in the study noted here. The data used here are cross‐sectional given specific questions about parenthood and sociodemographic characteristics. Following consent, participants completed a survey about their experiences as SM adults, which included questions about their demographic characteristics and experiences of discrimination among others. The Institutional Review Board at the University of California at Los Angeles approved the study. All other universities involved in this project completed a reliance agreement. For an in‐depth description of the methodology, see Meyer et al. (2020).

The three cohorts are designated as the *Pride* cohort, 52–59 years old at the time of data collection (born 1957–1964), who were adolescents during the Stonewall Riots; the *Visibility* cohort, ages 34–41 (born 1975–1982), who were adolescents during the AIDS crisis; and the *Equality* cohort, ages 18–25 (born 1991–1998), who came of age during marriage equality debates in the United States. Among all participants (*N* = 1502), parents were identified by a “Yes/No” response to, “Do you have any children?” Those who said, “Yes,” were categorized as parents (*n* = 297; 19.77%); those who said “No” (*n* = 1205; 80.23%) were categorized as childfree.

### 2.2 Measures

#### 2.2.1 Demographic Factors

Participants responded to questions about demographic characteristics, including sexual and gender identities, race/ethnicity, relationship status, partner gender, educational attainment, poverty status, geographic region, and urbanicity. Sexual identity options included bisexual, gay, lesbian, queer, same‐gender loving, straight/heterosexual, and a write‐in option. Gender identity options included woman, man, nonbinary/genderqueer, transgender man/female‐to‐male, and transgender woman/male‐to‐female. In study recruitment, participants who identified as transgender were redirected to a different transgender‐specific study; some participants who enrolled in the study because their primary identity was LGB also identified as transgender or another gender diverse identity at the time of study participation.

Race/ethnicity options included American Indian or Alaskan Native, Asian/Asian American, Black/African American, Hispanic/Latino/Spanish origin, Middle Eastern/North African, Native Hawaiian/Pacific Islander, and White. Participants were asked to select one option for sexual and gender identity questions with race/ethnicity being a check all that applied to the question. Relationship status was assessed via a Yes/No item (i.e., “Are you currently in a relationship or feel a special commitment to someone?”). Partner gender options were the same as participant gender identity options. Education was collapsed into four options of high school or less, some college, college, or beyond college. Geographic region included Midwest, Northeast, South, and West. Poverty status was a Yes/No variable developed by weighting Census estimates of poverty thresholds based on income. Urbanicity was a dichotomized item based on the USDA Rural–Urban Commuting Area coding system by zip code.

### 2.3 Data Analytic Plan

We conducted Rao Scott design‐adjusted *F* tests for our variables, as they were categorical (Rao and Scott 1984). We also conducted multivariate logistic regressions to assess the likelihood of being a parent relative to childfree SM adults. All analyses included survey weights to best generalize these findings to the broader US population of SM adults. We first report the descriptive information and statistical differences in sociodemographic characteristics between SM parents and childfree adults (Table 1). Second, we report the likelihood of SM adults being parents or not (Table 2). Finally, we report on the differences in sociodemographic characteristics among just SM parents, across the three cohorts (Table 3).

**TABLE 1:** Demographic characteristics of sexual minority parents and childfree adults.

	Parents *n* = 297 (%)	Childfree adults *n* = 1205 (%)	*F*(df)	*p*
Age cohort				
*Pride* (52–59) (*n* = 665)	146 (33.05)	322 (13.91)	*F*(1.83, 2740.64) = 60.82	< 0.001
*Visibility* (34–41) (*n* = 369)	118 (45.11)	251 (15.90)		
*Equality* (18–25) (*n* = 468)	33 (21.84)	632 (70.19)		
Sexual identity				
Lesbian/Gay	138 (36.78)	683 (48.67)	*F*(1.82, 2709.15) = 11.67	< 0.001
Bisexual	131 (55.57)	359 (37.72)		
Another identity	24 (7.65)	156 (13.62)		
Gender identity				
Cisgender woman	219 (79.34)	524 (50.07)	*F*(1.93, 2889.57) = 35.45	< 0.001
Cisgender man	66 (16.68)	599 (41.66)		
Nonbinary/Genderqueer	12 (3.97)	82 (8.27)		
Relationship status (% Yes)	237 (81.03)	682 (57.17)	*F*(1, 1489) = 43.90	< 0.001
Race/Ethnicity				
Black/African American	46 (15.95)	191 (16.54)	*F*(1.97, 2960.91) = 2.93	0.065
Latino/Hispanic	45 (15.19)	248 (22.39)		
White	206 (68.85)	766 (61.07)		
Educational attainment				
High school or less	46 (35.65)	261 (44.26)	*F*(2.69, 4036.24) = 6.22	< 0.001
Tech/trade or some college	96 (33.23)	390 (31.36)		
College	71 (14.43)	354 (16.26)		
Beyond college	84 (16.69)	200 (8.12)		
Geographic region				
Midwest	48 (20.51)	222 (20.10)	*F*(2.94, 4417.08) = 0.34	0.796
Northeast	63 (21.53)	236 (18.49)		
South	99 (32.94)	410 (34.85)		
West	87 (25.03)	337 (26.56)		
Poverty status (% Yes)	54 (30.49)	153 (16.62)	*F*(1, 1483) = 10.29	< 0.001
Urbanicity (% Urban)	239 (77.96)	1062 (88.96)	*F*(1, 1479) = 9.18	< 0.001
Partner gender				
Cisgender woman	102 (38.37)	219 (29.36)	*F*(3.60, 3304.35) = 1.84	0.113
Cisgender man	128 (59.21)	438 (66.92)		
Nonbinary/Genderqueer	1 (1.51)	3 (1.58)		
Transgender woman	2 (0.43)	10 (0.41)		
Transgender man	4 (0.49)	11 (1.73)		

*Note:* Raw n's are presented in the table with weighted percentages included in parentheses.

**TABLE 2:** Multivariate logistic regression: probability of being a parent (*n* = 297) versus being childfree (*n* = 1205).

	OR (95% CI)	*p*
Cohort (ref = “Visibility (34–41)”)		
Equality (18–25)	0.07 (0.04, 0.11)	< 0.0001
Pride (52–59)	1.54 (0.97, 2.45)	0.070
Gender (ref = “Men”)		
Woman	6.33 (4.05, 9.90)	< 0.0001
Nonbinary/Genderqueer	3.54 (1.42, 8.83)	0.007
Sexual identity (ref = “Gay/Lesbian”)		
Bisexual	3.37 (2.19, 5.18)	< 0.0001
Other	1.16 (0.57, 2.34)	0.687
Race (ref = “White”)		
Black/African American	1.12 (0.68, 1.84)	0.651
Latino	1.03 (0.63, 1.69)	0.893
Relationship status (ref = “Single”)		
Civil union/partnership	8.98 (2.96, 27.22)	0.0001
Not married	2.00 (1.30, 3.09)	0.002
Education (ref = “Beyond college”)		
High school or less	2.07 (1.15, 3.73)	0.016
Some college	0.88 (0.48, 1.63)	0.675
Tech/trace or some college	1.49 (0.84, 2.62)	0.172
Geographic region (ref = “Northeast”)		
Midwest	1.22 (0.71, 2.09)	0.468
South	1.07 (0.65, 1.75)	0.795
West	0.93 (0.55, 1.56)	0.784

**TABLE 3:** Sociodemographic characteristics across cohorts among parents.

	Pride (*n* = 146)	Visibility (*n* = 118)	Equality (*n* = 33)	*F*(df)	*p*
Sexual identity					
Lesbian/Gay	98 (66.74%)	34 (23.99%)	6 (17.93%)	*F*(3.50, 1051.26) = 11.02	< 0.001
Bisexual	39 (29.08%)	68 (64.97%)	24 (76.03%)		
Another identity	7 (4.18%)	14 (11.04%)	3 (6.04%)		
Gender identity					
Cisgender woman	89 (60.58%)	100 (87.32%)	30 (91.52%)	*F*(1.99, 522.22) = 10.39	< 0.001
Cisgender man	51 (35.37%)	15 (10.98%)	0 (0%)		
Nonbinary/Genderqueer	6 (4.05%)	3 (1.70%)	3 (8.48%)		
Relationship status (% Yes)	109 (72.94%)	101 (84.94%)	27 (85.27%)	*F*(1.98, 582.61) = 2.04	0.131
Race/Ethnicity					
Black/African American	15 (7.62%)	20 (16.90%)	6 (26.59%)	*F*(3.38, 999.19) = 1.70	0.160
Latino/Hispanic	24 (18.70%)	16 (13.18%)	10 (14.06%)		
White	107 (73.68%)	82 (69.92%)	17 (59.35%)		
Educational attainment					
High school or less	18 (26.40%)	17 (32.95%)	11 (55.24%)	*F*(3.91, 1156.31) = 3.84	0.005
Some college	40 (28.53%)	39 (34.42%)	17 (37.87%)		
College	39 (20.92%)	29 (14.79%)	3 (3.88%)		
Beyond college	49 (24.15%)	33 (17.84%)	2 (3%)		
Geographic region					
Midwest	18 (10.28%)	24 (28.00%)	6 (20.50%)	*F*(5.29, 1564.56) = 1.85	0.096
Northeast	34 (29.64%)	25 (17.76%)	4 (17.05%)		
South	51 (30.66%)	32 (28.76%)	16 (45.01%)		
West	43 (29.42%)	37 (25.48%)	7 (17.44%)		
Poverty status (% Yes)	14 (12.85%)	23 (29.40%)	17 (59.09%)	*F*(1.60, 469.90) = 8.82	< 0.001
Urbanicity (% Urban)	116 (82.31%)	101 (83.43%)	22 (60.23%)	*F*(1.55, 454.12) = 3.35	0.048
Partner gender					
Cisgender woman	61 (56.90%)	36 (36.63%)	5 (17.92%)	*F*(3.43, 810.66) = 4.08	0.005
Cisgender man	47 (42.33%)	61 (60.57%)	20 (78.29%)		
Nonbinary/Genderqueer	1 (0.77%)	2 (1.76%)	1 (1.94%)		
Transgender woman	—	—	1 (1.85%)		
Transgender man	—	2 (1.04%)	—		

Although similar, *F* tests and multivariate logistic regressions were both conducted as a way of providing a comprehensive understanding of the demographic characteristics of SM parents and childfree adults. Rao Scott design‐adjusted *F* tests denote nuances between observed and expected frequencies for SM parents and childfree adults but is limited in that specific effect sizes are not present to interpret differences between specific groups. Multivariate logistic regressions address the limitations of Rao Scott design‐adjusted *F* tests by providing interpretable odds ratios that can indicate differences in likelihood of parent status but are limited in that a reference group must be specified. Thus, we present Rao Scott design‐adjusted *F* tests to ensure that differences in distributions are not obscured due to the selection of a reference group, while also including multivariate logistic regressions to assess likelihood‐based differences but are limited due to the determination of a reference group.

## 3 Results

### 3.1 Differences in Sociodemographic Characteristics Based on Parent Status

In assessing weighted percentages, slightly more than half of all parents identified as bisexual (55.57%), approximately one‐third identifying as lesbian or gay (36.78%), with less than 10% of parents identifying with another sexual identity (7.65%). This pattern for sexual identity did not occur for childfree adults, where slightly less than half of childfree adults identified as lesbian or gay (48.67%). A substantial majority of parents identified as cisgender women (79.34%), followed by slightly more than 10% of parents identifying as cisgender men (16.68%), and a small group of those who identified as nonbinary/genderqueer (3.98%). This pattern for gender identity also did not occur for childfree adults, where half of all adults identified as cisgender women (50.07%). As it pertains to relationship status, there was again a large difference between parents and childfree adults, such that a substantial majority of parents reported being in a relationship (81.03%), whereas among childfree adults slightly more than half were in a relationship (57.17%). When considering partner gender, among parents, cisgender women were approximately equally split between being partnered with another cisgender woman (47.13%) or a cisgender man (50.00%), with very few being partnered with gender diverse (i.e., transgender man or woman, nonbinary/genderqueer) individuals (2.27%). Furthermore, among parents who were cisgender men, almost three‐quarters (71.15%) were partnered with another cisgender man, a smaller number partnered with a cisgender woman (26.92%), and only one participant in our sample being partnered with a transgender man. Finally, for those who identified as nonbinary or genderqueer and were partnered, most reported that their partner was a cisgender woman (44.44%) or man (33.33%), with fewer being partnered with another nonbinary or genderqueer person (22.22%). Similar patterns emerged in partner gender for childfree adults.

Related to race/ethnicity, almost three‐quarters of parents were White (68.85%) which was a slightly greater proportion relative to childfree adults (61.07%). However, although the number of Black/African American and Latino/Hispanic parents were approximately equal (15.95%, 15.19%, respectively), this was not the case for childfree adults where there were slightly greater numbers of Latino/Hispanic (22.39%) than Black/African American adults (16.54%). Regarding educational attainment, approximately one‐third of parents reported having a high school degree or less (35.65%), with a similar number reporting a technical/trade school or some college education (33.23%). Approximately equal numbers of parents reported a college (14.43%) or greater than college degree (16.69%) both of which represented about 15% of the sample. There was an equal distribution of parents and childfree adults across the United States, such that more than one‐third of all adults lived in the Southern United States (32.94% and 34.85% for parents and childfree adults, respectively), with all other geographic regions representing one‐fifth to a quarter of participants (i.e., 18.49%–26.56% regardless of parent status). Furthermore, a greater proportion of childfree adults lived in urban areas (88.96%) compared with parents (77.96%). Finally, almost a third of all parents reported living at or below the poverty line (30.49%), which is almost twice the rate of living at or below the poverty line for childfree adults (16.62%).

We found several significant differences in sociodemographic characteristics in our sample based on parent status. To begin, there was a significant association with parent status by cohort, *F*(1.83, 2740.64) = 60.82, *p* < 0.001. A smaller proportion of parents belonged to the *Equality* cohort (18–25 years old; 21.84%) than the *Visibility* (34–41 years old; 45.11%), and *Pride* (52–59 years old; 33.05%) cohorts. Furthermore, there was a significant association in sexual identity based on parent status, *F*(1.82, 2709.15) = 11.67, *p* < 0.001, such that there was a greater proportion of bisexual parents than bisexual childfree adults. There was also a significant association in gender identity based on parent status, *F*(1.93, 2889.57) = 35.45, *p* < 0.001, such that a greater proportion of parents were cisgender women than childfree adults. In addition, there was a significant difference in relationship status, *F*(1, 1489) = 43.90, *p* < 0.001, such that a greater proportion of parents were in relationships than childfree adults.

We also found a significant association in educational attainment, *F*(2.69, 4036.24) = 6.22, *p* < 0.001, such that parents had greater levels of educational attainment than childfree adults. Furthermore, there was a significant association in poverty status, *F*(1, 1483) = 10.29, *p* < 0.001, such that a greater proportion of parents were living at or below the poverty line compared with childfree adults. In addition, there was a significant association in urbanicity, *F*(1, 1479) = 9.18, *p* < 0.001, such that a greater proportion of childfree adults lived in urban areas when compared with parents. Finally, we found no significant associations in race/ethnicity, *F*(1.97, 2960.91) = 2.93, *p* = 0.065, geographic region, *F*(2.94, 4417.08) = 0.34, *p* = 0.796, or partner gender, *F*(3.60, 3304.35) = 1.84, *p* = 0.113, based on parent status. See Table 1 for omnibus test statistics.

The logistic regression analyses conducted also suggested several significant associations between demographic characteristics and likelihood of being a parent compared with being childfree. Individuals in the *Equality* cohort were lower the odds of being a parent (than childfree) relative to the *Visibility* cohort, but there was no significant association in likelihood of parent status for the *Pride* cohort relative to the *Visibility* cohort. Furthermore, relative to cisgender men, cisgender women and nonbinary/genderqueer individuals were higher the odds of being a parent than childfree. Bisexual individuals had higher odds of being a parent than childfree relative to lesbian/gay individuals, but there was no significant association in likelihood of parent status for those in the ‘other’ sexual identity group.

All individuals in relationships, relative to being single, had higher odds of being a parent than childfree. Individuals who had a high school degree (or equivalent) or less were also higher the odds of being a parent relative to those with a graduate degree (associations with other levels of educational attainment were not significant). Finally, we found no differences based on race/ethnicity or geographic region as it pertains to the likelihood of being a parent than childfree SM adult (relative to being White or living in the Northeastern United States; see Table 2 for statistics).

### 3.2 Demographic Characteristics of SM Parents by Cohort

There was also a significant association between cohort and sexual identity among parents, *F*(3.50, 1051.26) = 11.02, *p* < 0.001. More than half of parents in the *Pride* cohort (52–59 years old) identified as lesbian or gay (66.74%), whereas bisexual parents were most represented in the *Visibility* (34–41 years old; 64.97%) and *Equality* cohorts (18–25 years old; 76.03%). There was also a significant association between cohort and gender identity among parents, *F*(1.99, 522.22) = 10.39, *p* < 0.001. Among the *Pride* cohort (52–59 years old), closer to half of parents identified as cisgender women (60.58%), whereas the majority of parents in the *Visibility* (34–41 years old; 87.32%) and *Equality* (18–25 years old; 91.52%) cohorts were cisgender women.

Most parents across cohorts were partnered: almost three‐quarters of the *Pride* cohort (52–59 years old; 72.94%), and even more in the *Visibility* (34–41 years old; 84.94%) and *Equality* (18–25 years old; 85.27%) cohorts. There was no significant association between cohort and relationship status among parents, *F*(1.98, 582.61) = 2.04, *p* = 0.131. However, there was a significant association between cohort and partner gender, *F*(3.43, 810.66) = 4.08, *p* = 0.005, such that there was a greater proportion of non‐cisgender partners in the younger cohorts and a greater balance of partners being cisgender women or men in the *Pride* cohort.

In the *Pride* cohort (52–59 years old), more than half of parents were partnered with cisgender women (56.9%), followed by those partnered with cisgender men (42.33%), and a much smaller group with a nonbinary/genderqueer partner (0.77%). Among the *Visibility* cohort (34–41 years old), most parents were partnered with cisgender men (60.57%), followed by cisgender women (36.63%), nonbinary/genderqueer individuals (1.76%), or transgender men (1.04%). Finally, among the *Equality* cohort (18–25 years old), most parents reported that their partners were cisgender men (78.29%), with smaller groups partnered with cisgender women (17.92%), nonbinary/genderqueer individuals (1.94%), or transgender women (1.85%).

There was no significant association between cohort and racial/ethnic identity among parents, *F*(3.38, 999.19) = 1.70, *p* = 0.160. Regarding education, many parents reported some college (28.53% *Pride*; 34.42% *Visibility*; 37.87% *Equality*), followed by a high school degree (or equivalent) or less (26.4% *Pride*; 32.95% *Visibility*; 55.24% *Equality*), an advanced degree (24.15% *Pride*; 17.84% *Visibility*; 3% *Equality*), and finally a bachelor's degree (20.92% *Pride*; 14.79% *Visibility*; 3.88% *Equality*). There was a significant difference across cohorts in educational attainment, *F*(3.91, 1156.31) = 3.84, *p* = 0.005 such that the *Pride* and *Visibility* cohorts had greater educational attainment than the *Equality* cohort.

There was a significant association between poverty status and cohort among parents, *F*(1.60, 469.90) = 8.92, *p* < 0.001. The proportion of parents living at or below the poverty line increased among the younger cohorts (12.85% *Pride*; 29.4% *Visibility*; 59.09% *Equality*). We also found a significant association between urbanicity and cohort, *F*(1.55, 454.12) = 3.35, *p* = 0.048, such that a greater proportion of the older cohorts (82.31% *Pride*; 83.43% *Visibility*) lived in urban areas relative to the *Equality* cohort (60.23%). Finally, we found no differences between geographic region and cohort among parents, *F*(5.29, 1564.56) = 1.85, *p* = 0.096.

## 4 Discussion

We used data from the first national probability‐based study on the experiences of SM people to investigate demographic characteristics between SM parents and childfree adults across three cohorts. There were significant differences in demographic characteristics between SM parents and childfree adults as well as just parents across cohorts, in addition to differences in likelihood of being a parent. There was a greater proportion of SM parents than childfree adults who identified as bisexual, were cisgender women, in a relationship, had generally greater educational attainment, lived at or below the poverty line, or lived in a rural area. Additionally, there were differences in likelihood of being a parent, rather than childfree, across most demographic characteristics, excluding race/ethnicity and geographic region. Furthermore, we found that among parents across cohorts there were differences in sexual and gender identity, partner gender, educational attainment, poverty status, and urbanicity, but not relationship status, race/ethnicity, or geographic region. These findings represent an important contribution to the family sciences as previous research has largely focused on caregivers with minors, rather than SM parents more broadly (i.e., parents with children over the age of 18 years old; Gates 2015).

Our findings related to demographic characteristics generally align with previous literature on SM parents and childfree adults indicating that disparities exist within SM communities including greater poverty status or lower educational attainment among parents (Gates 2013, 2015). This work also aligns with research that has found a greater proportion of parents among bisexual or cisgender women, those in different‐sex relationships (Goldberg and Kerith 2018), or those living in rural areas (Reczek 2020; Stone 2018). These findings also suggest a possible shift in SM parenthood related to race/ethnicity and geographic region: we find no significant differences in these sociodemographic characteristics, while past work suggests a greater proportion of racial/ethnic minorities and SM parents living in the Southern or Midwestern United States (Gates 2013, 2015).

There are several ways in which we might understand the discrepancies between the findings presented here and prior research. For example, methodological approaches and sampling used in previous work (e.g., Gates 2013, 2015) were distinct from the national probability approach utilized here. Previous research used national datasets which may have been limited in that they assessed differences between same‐ and different‐sex couples, unintentionally erasing SM people in different‐sex relationships or who were single. Nuances such as identification of participants which might include asking about same‐sex attraction (or behavior; Riskind and Tornello 2017) as opposed to identity or assessing whether a child is currently in the home compared with having ever had a child could also explain our findings.

Another possibility could be related to the age range of our sample. Research has found that SM parents who had children in previous different‐sex relationships were more likely to also be people of color and would be classified in older cohorts (Gates 2015; Reczek 2020). Thus, as sexual intercourse in a previous different‐sex relationship prior to one's LGBTQ+ identification has decreased in recent decades, so too might racial/ethnic differences (Reczek 2020). Greater representation of younger individuals, as is the case in our sample, could potentially be an indication of a shift in racial/ethnic and geographic differences in younger generations of SM parenthood which has been suggested in prior research (Gates 2015). Furthermore, the absence of some racial/ethnic groups (e.g., Asian Americans) and differences in overall sample characteristics could explain our findings. Previous work by Wilson and Bouton (2024) found that same‐sex couples who are parents were more likely to be people of color than White. This research also indicated that racial/ethnic differences in parenthood status was primarily among parents who were assigned male at birth, Black, and partnered. However, our sample had fewer cisgender men overall, and a greater percentage of cisgender women who were parents compared with the work by Wilson and Bouton (2024). Thus, differences in racial/ethnic sample make‐up, a greater proportion of parents who were cisgender women, and a general absence of transgender parents, may have played a role in our findings.

Among SM parents, the *Visibility* (middle; 34–41 years old) cohort represented the greatest proportion of parents, followed by the *Pride* (oldest; 52–59 years old) and then *Equality* (youngest; 18–25 years old) cohorts, and there were differences in educational attainment which provides mixed support for our initial expectations. We anticipated that older cohorts would have increased numbers of parents. This was not the case, however, as the *Visibility* cohort had a greater proportion of parents relative to the *Pride* and *Equality* cohorts. Parents in the *Equality* cohort may have come of age during a period in which multiple pathways to parenthood were increasingly available (e.g., advancement of ART and adoption law; Jain et al. 2019; Wexler et al. 2023). In contrast, parents in the *Pride* and *Visibility* cohorts may have become parents prior to the increasing availability of pathways to parenthood and many in the *Equality* cohort may intend to become parents but have not chosen a preferred pathway to parenthood.

We also found that a greater proportion of parents in the *Pride* cohort were lesbian/gay and/or cisgender women relative to the younger cohorts. One explanation for the greater proportion of lesbian/gay parents in the *Pride* cohort relative to a greater proportion of bisexual parents in the younger cohorts is the cultural and political climate when these parents came of age. Parents who identify as lesbian/gay in the *Pride* cohort may have at one point identified as bisexual or had children in the context of a presumed cisgender, heterosexual relationship and later identified as lesbian/gay, whereas younger individuals may feel more comfortable openly identifying as bisexual given an improved political/social climate in the United States. An additional interpretation is that more parents lived in rural areas, which also tends to have individuals with lower educational attainment for a number of reasons (e.g., structural poverty) and in turn, fewer parents had the opportunity to receive an advanced education.

Although fewer in number, a greater proportion of parents in the *Equality* (i.e., youngest) cohort were living at or below the poverty line and in rural areas relative to the *Visibility* and *Pride* cohorts. Although research has found that younger SM adults are more likely to be living at or below the poverty line compared with their cisgender heterosexual counterparts (Badgett et al. 2019), this may be one of the first studies to document this among SM parents. That a greater proportion of parents in the *Equality* cohort lived in rural areas also seems to be an explanation for differences in urbanicity between parents and childfree adults, as parents in older cohorts lived in urban areas equivalent to childfree adults. Research suggests that SM parents who live in rural areas may lack support and resources related to their SM identity more so than SM people who live in urban areas (Stone 2018). Poverty status and urbanicity then, may magnify potential risk of experiencing future health disparities among younger SM parents as our findings suggest that they are also living in rural areas, which is associated with living at or below the poverty line (Gates 2015; Reczek 2020). While some of these differences, such as in poverty status or education, have been studied, research on the demographic characteristics among same‐gender or SM couples have been mixed. Some research has found that, despite SM individuals in same‐gender relationships having greater educational attainment and income overall, SM parents in same‐gender relationships report lower education and income relative to cisgender heterosexual parents (and higher poverty rates; Smock and Schwartz 2020). Thus, our findings indicate that this trend of higher poverty rates persist even among younger generations of SM parents.

We found no significant associations between race/ethnicity, geographic region, or relationship status among parents based on cohort status. The lack of significant associations in race/ethnicity and geographic region matches our findings when comparing SM parents and childfree adults. We also found that there were no significant differences based on relationship status among parents based on cohort status. This lack of association is likely due to the overrepresentation of SM parents being partnered, if the majority of SM parents are partnered then there may be no differences in relationship status across cohorts. Given that a long‐standing relationship may provide several benefits, especially a relationship with legal recognition (e.g., marriage), research should investigate single SM parents as they may report different sociodemographic characteristics or be at elevated risk for negative outcomes that are reduced by relationship status (e.g., partnered SM adults report reduced stress; Gates 2015; Reczek 2020).

Although there was no association in relationship status among parents, there was a significant difference based on partner gender such that more parents were in a relationship with cisgender men. This difference is likely explained by three factors. First, the proportion of parents that were partnered with cisgender women steadily decreased across younger cohorts. Second, there were more bisexual parents among the younger cohorts who were women, thus increasing the likelihood of cisgender male partners. Third, there was a larger proportion of non‐cisgender partners among the younger cohorts which contributed to this significant difference. One possibility is that having a biological child through sexual intercourse is still a common pathway to parenthood, but SM people are no longer doing so in heterosexual relationships. Instead, cisgender bisexual women are most represented among younger cohorts because they have the option to have a child with cisgender male partners (or those assigned male at birth), whereas other pathways to parenthood may be too expensive for young people.

This national probability study of SM people in the United States does have several limitations. Future work should include transgender/nonbinary adults and Asian Americans. Although respondents whose primary LGBT identity was transgender were routed to a different transgender‐specific study, there were several participants in this study who identified as nonbinary or genderqueer and not necessarily as transgender. Research should investigate potential differences in sociodemographic characteristics and experiences of those who identify as nonbinary but not transgender. Investigation into the pathway to parenthood is also of interest but outside the scope of these data as small sample sizes precluded analyses. Furthermore, given the data structure we could not fully account for age in our analyses. However, this work has substantial strengths, namely that there is little work that has investigated the sociodemographic characteristics of SM parents and childfree adults, in the same sample, across time and thus our research may be the first to report on SM parent backgrounds in this way. Additionally, use of a national probability sample provides a more comprehensive view of SM people's sociodemographic characteristics than other sampling approaches (Reczek 2020).

## 5 Conclusion

Our findings reported here suggest that there are important changes in sociodemographic characteristics not only between parents and childfree adults but also among parents across three generations of SM adults. Our results indicate an ongoing change in the gender of parents and the gender of parents' partners, as well as in educational attainment among SM parents relative to childfree adults. Furthermore, of particular concern is that SM parents report greater rates of poverty than their childfree counterparts, especially among the youngest cohort of SM parents in our sample. Finally, some of our findings also align with previous research such as greater proportions of bisexual women being parents relative to other SM identities, and especially among younger SM adults. Researchers and providers in family planning settings must be aware of the continued shifts in family sociodemographic characteristics over time such as poverty status and the role of identity in the context of parenthood, particularly for bisexual parents.

## Conflicts of Interest

The authors declare no conflicts of interest.

## Disclaimer

The content is solely the responsibility of the authors and does not necessarily represent the official views of the National Institutes of Health.
